# Comparative analysis of the profiles of IgG subclass-specific responses to *Plasmodium falciparum* apical membrane antigen-1 and merozoite surface protein-1 in naturally exposed individuals living in malaria hypoendemic settings, Iran

**DOI:** 10.1186/s12936-015-0547-0

**Published:** 2015-02-05

**Authors:** Maryam Rouhani, Sedigheh Zakeri, Akram A Mehrizi, Navid D Djadid

**Affiliations:** Malaria and Vector Research Group (MVRG), Biotechnology Research Center (BRC), Pasteur Institute of Iran, Pasteur Avenue, P.O. BOX 1316943551, Tehran, Iran

**Keywords:** Malaria, *P. falciparum*, AMA-1, MSP-1_19_, Sero-epidemiology, Vaccine

## Abstract

**Background:**

*Plasmodium falciparum* apical membrane antigen-1 (PfAMA-1) and the 19-kDa C-terminal region of merozoite surface protein-1 (PfMSP-1_19_) are candidate malaria vaccine antigens expressed on merozoites and sporozoites. This investigation was performed to evaluate simultaneously the naturally-acquired antibodies to PfAMA-1 and PfMSP-1_19_ and to compare IgG subclass profiles to both antigens in naturally exposed individuals living in malaria hypoendemic areas in Iran to determine which antigen has better ability to detect sero-positive individuals infected with *P. falciparum*.

**Methods:**

In this investigation, 101 individuals from the malaria-endemic areas in Iran were examined. PfAMA-1 and PfMSP-1_19_ were expressed in *Escherichia coli*, and IgG isotype composition of naturally acquired antibodies to the antigens (as single or in combination) was measured by ELISA assay.

**Results:**

The result showed that 87.1% and 84.2% of the studied individuals had positive anti-PfAMA-1 and -PfMSP-1_19_ IgG antibody responses, respectively, and the prevalence of responders did not differ significantly (*P* > 0.05). Moreover, IgG1 and IgG3 were predominant over IgG2 and IgG4 antibodies and the prevalence of IgG and its subclasses to two tested antigens had no significant correlation with age and exposure (*P* > 0.05). The present data confirmed that when recombinant PfAMA-1 and recombinant PfMSP-1_19_ antigens were combined in ELISA at equal ratios of 200 ng (100 ng each antigen/well) and 400 ng (200 ng each antigen/well), 86.1% and 87.1% of positives sera were detected among the examined samples, respectively.

**Conclusions:**

The two tested recombinant antigens are immunogenic molecules, and individuals in low transmission areas in Iran could develop and maintain equal immune responses to PfAMA-1 and PfMSP-1_19_. Therefore, these results could support the design of a universal PfAMA-1- and PfMSP-1_19_-based vaccine. Also, both recombinant antigens could be used in combination as reliable serology markers to perform immuno-epidemiological studies in malaria-endemic areas of Iran during elimination strategy. The present information could be of use in control and elimination programmes in Iran and other similar malaria settings.

## Background

Malaria is caused by different obligate intracellular parasites. *Plasmodium falciparum* is one the most lethal species of malaria parasites that infects humans [1]. This parasite species is responsible for most of the pathology associated with the disease [2]. The unacceptable health burden of malaria and its economical and social impacts have led to making a plan for scaling-up malaria control, elimination, and global eradication [3]. However, the hopes of achieving this goal are diminishing due to the limited effective control tools, the emergence and rapid widespread occurrence of drug-resistant parasites, and the resistance of mosquitoes to insecticides.

Therefore, a search for new tools is required to control or eliminate malaria. One of the effective tools to combat infectious diseases is vaccination [4]. Hence, to design an efficient malaria vaccine, it is essential to determine the key target antigen that induces protective immunity for applying in vaccine development [5].

Immuno-epidemiological studies in diverse malaria-endemic regions with different level of transmission and human genetic background provide more information to understand the host immune response to *P. falciparum*, and also it may help to design an effective vaccine against this species. For instance, individuals who are living in endemic areas are simultaneously and repeatedly challenged with numerous malaria antigens. In high transmission regions, all individuals have many infections during their life; therefore, protective immunity develops with age/exposure in these individuals [6]. In contrast, in low and unstable transmission regions, there is a lack of such correlation with age [7-12].

A passive transfer study conducted in the 1960s showed that IgG antibody is a major component of naturally-acquired protective immune responses of *P. falciparum* [13,14]. In malaria-endemic areas, older children and adults develop naturally-acquired immunity to malaria but remain susceptible to infection.

In the life cycle of human malaria parasites, the invasion of erythrocytes by merozoites (the only extracellular stage of the asexual cycle) is an obligatory step during blood-stage infection, and blocking this step with antibodies would lead to hinder the invasion of red blood cells [13,15,16]. The proteins that are present on the surface of invasive merozoites of *Plasmodium* are essential targets for development of an effective malaria vaccine. Among them, merozoite surface protein-1 (MSP-1) and apical membrane antigen-1 (AMA-1) are considered leading and attractive malaria blood-stage vaccine candidate antigens [17-21]. These two antigens are located on the merozoite surface and undergo proteolytic processing before the invasion of merozoite into the red blood cells.

AMA-1 is a type I integral membrane protein expressed on merozoites and sporozoites and initially located in the micronemes [22-25]. AMA-1 is synthesized in segmenting schizonts as an 83-kDa precursor protein. At about the time of merozoite release and erythrocyte invasion, the prodomain is cleaved to a 66 kDa membrane-bound form [26,27], where it is subsequently shed as 44- and 48-kDa forms [27,28]. This protein has three subdomains defined by their disulfide bonds [29] and contains 16 conserved cysteine residues forming eight intra molecular disulfide bonds [26]. Furthermore, individuals living in areas where malaria is endemic have antibodies against AMA-1 [30-32], and these antibodies efficiently inhibit the process of red blood cells invasion *in vitro* [28,31,33]. The protective efficacy of AMA-1-based vaccines against parasite challenge has been demonstrated in many rodent and monkey models [22,34,35].

MSP-1 is synthesized as a 195-kDa protein and sequentially processed into a cysteine-rich 19-kDa fragment (MSP-1_19_) [36]. This protein contains two epidermal growth factor (EGF)-like domains [37,38]. Several *in vitro* and *in vivo* studies have shown that the PfMSP-1_19_ is an ideal target for blocking parasite invasion into the erythrocyte [39-43]. Antibodies to PfMSP-1_19_ are found in the majority of malaria-exposed individuals from endemic areas [44,45], and these antibodies correlate with the development of clinical immunity against *P. falciparum* malaria [44,46].

In Iran, malaria is hypoendemic with seasonal transmission. In 2013, due to elimination strategies, about 1,373 malaria cases were reported from Iran that more than 80% of these cases were *Plasmodium vivax* and the rest of them were *P. falciparum* (the Ministry of Health, 2013, unpublished). In this area, there is no record of severe malaria or death due to malaria. Most of the patients are adults and may experience several infections by *P. falciparum* and *P. vivax* with clinical symptoms. As a continuation of the previous immuno-epidemiological studies in Iran [10,11,47-49], in the present study, the main objective was to evaluate simultaneously the naturally acquired antibodies responses to two recombinant proteins of *P. falciparum* (PfMSP-1_19_ and PfAMA-1) among falciparum malaria subjects in the hypoendemic areas of Iran.

These two antigens were selected for this study because the evidence showed that there is likely a association between the presence of antibodies to these antigens and protection [50,51]. In fact, it demonstrates that both antigens are potential asexual erythrocytic stage vaccine candidates. Therefore, the main objective of the present work was to evaluate and compare the profile of IgG subclass-specific responses to PfAMA-1 and PfMSP-1_19_ in naturally exposed individuals living in the malaria hypoendemic areas, Iran. Also, the association between naturally acquired anti-PfAMA-1 and -PfMSP-1_19_ isotype responses and host age and exposure was assessed in this study. Furthermore, as both target antigens are used as a mean of detection of antibody responses in areas of low endemicity [52-55]; therefore, the second objective of the present study was to determine which antigen has better ability to detect sero-positive individuals infected with *P. falciparum*. The current information could be of value for control and elimination programmes in areas of low endemicity.

## Methods

### Study area, subjects, and blood sample collection

This study was carried out in Chabahar, Sistan and Baluchistan Province in south-eastern Iran. In this area, most of the patients are adults and may experience several infections by *P. falciparum* and *P. vivax*. In this investigation, 101 blood samples were obtained from suspected patients attended at the Malaria Health Center in Chabahar Public Health Department in Sistan and Baluchistan Province from May 2006 to 2012. Before blood collection, an informed consent was obtained from adults or parents or legal guardians of children who were participant in this study. The diagnosis of malaria was made by microscopic examination of blood smears stained with Giemsa. All *P. falciparum*-positive samples were verified by molecular diagnosis using the 18ssrRNA gene as described previously [56]. The control blood samples (n = 30) were obtained from the residents in Tehran (Iran) with no known pervious exposure to malaria. From all subjects, 2 ml venous blood was collected for both Plasmodium DNA detection and serum collection in EDTA tubes. The collected blood samples were transported on-ice to the main laboratory in the Institut Pasteur Iran. The majority of the patients were male (75.2%) with a mean age of 29.9 ± 12.6 years (ranged between 4 to 75 years old). The patients’ travel histories were obtained by a physician prior to sampling. The demographic information of the examined groups is shown in Table 1. This study was approved by the Ethical Review Committee of Research in Institut Pasteur Iran.

**Table 1:** **Demographic characteristics of the examined subjects in this study**

**Mean age ± SD**	**Sex (%)**		**Exposure (%)**		**Nationality (%)**			**Travel history (%)**	
	**Male**	**Female**	**First exposure**	**More than one exposure**	**Iranian**	**Pakistani**	**Afghani**	**Yes**	**No**
29.9 ± 12.6	75.2	24.8	48.5%	51.5%	48.5	44.5	6.9	49.5	50.5

### Cloning and sub-cloning of PfAMA-1

Parasite genomic DNA was prepared from the whole blood by using the commercially available DNA Purification Kit (Promega, Madison, WI, USA). The DNA was dissolved in 30 μl TE buffer (10 mM Tris–HCl, pH 8.0, 0.1 mM EDTA) and kept at −20°C until use. In this study, for the expression of recombinant PfAMA-1 (rPfAMA-1), DNA samples with known sequences of PfAMA-1 (GenBank accession no. KC413989) were selected based on previous study [57]. Amplification of PfAMA-1 fragment corresponding to amino acids 96–542 (nucleotides: 286–1626) was performed using the following primers:

PfAMA-1-AF:AGCGGA*GGATCC*AGCATTGAAATAGTAGAAAGAAG, *Bam*HI site (italic)

PfAMA-1-AR: AGGGCC*AAGCTT*CATAAGTTGGTTTATGTTCAG, *Hind*III site (italic)

The PCR was performed at 95°C for 5 min, 30 cycles at 94°C for 1 min, 60°C for 1 min, 72°C for 1 min, followed by 60°C for 2 min and final extension at 72°C for 30 min. The PCR products were analysed by electrophoresis on 1% agarose gel under an ultraviolet light and purified by QIA quick Gel Extraction Kit (Qiagen, Germany). The gel-purified PCR products were cloned into pGEM-T Easy Vector (Promega, USA) and transformed into *Escherichia coli* DH5α. The transformed clones were selected on the Luria-Bertani agar medium, containing 100 μg/ml ampicillin, 1.5 mM isopropyl-β-D-thiogalactopyranoside (IPTG), and 0.04% X-gal. Positive clones were confirmed by plasmid isolation, followed by digestion with *Eco*RI, and the cloned fragments were then sequenced. Fragments corresponding to the PfAMA-1 sequence were excised with restriction enzymes (*Bam*HI and *Hind*III) and ligated to the *Bam*HI-*Hind*III sites of vector pQE-30 (Qiagen, Germany), which provides a poly-histidine (6-His) tag in N-terminus to facilitate further purification. The ligation mixtures were transformed into competent *E. coli* DH5α cells, and the recombinant clones were selected on ampicillin plates. The open reading frame was confirmed by sequencing, and this construct was used to transform *E. coli* M15 (pREP4) expression host (Qiagen, Germany).

### Expression and purification of rPfAMA-1

rPfAMA-1 was expressed in *E. coli* M15. Briefly, overnight cultures from single colonies of PfAMA-1-specific *E. coli* were expanded in TB (Terrific broth; pH 7.2), containing ampicillin (100 μg/ml) and kanamycin (25 μg/ml) with shaking (150 rpm) at 37°C until an optical density (OD) of 0.6 to 0.7 at 600 nm was reached. The expression of PfAMA-1 was induced with 0.5 mM IPTG (Sigma, USA). The culture was further grown for 4 h, and the *E. coli* cells were harvested by centrifugation and kept in −80°C until use. PfAMA-1 was expressed in inclusion bodies, and the cell pellet was dissolved in denaturation buffer (8 M Urea, 30 mM imidazole, 20 mM Tris–HCl, and 1 M NaCl, pH 7.9). The cells were lysed on ice by sonication (Ultraschallprozessor, Germany) with 10 cycles, each consisting of 20- second (s) pulses with 20-s intervals. The bacterial lysate was centrifuged at 14,000 × rpm at 4°C for 30 min. The supernatant was incubated with Ni^2+^-nitrilotriacetic acid agarose resin (Ni-NTA Agarose, Qiagen, Germany) at 4°C for 2 h, and the resin was packed into a column and was washed with a 10-column volume of wash buffer (6 M urea, 20 mMTris-HCl, 1 M NaCl, and 60 mM imidazole, pH 7.9). The bound protein was eluted with a buffer, containing 4 M urea, 200 mM imidazole, 20 mM Tris–HCl, and 300 mM NaCl, pH 7.9. The fractions containing PfAMA-1 was desalted with Econo-Pac 10DG columns (BioRad, USA) according to the manufacture’s manual and then concentrated with a concentrator (Eppendorf, Germany). The eluted proteins were analysed under reducing (with 1% SDS and 2% β-mercaptoethanol [2ME]) and non-reducing conditions (with SDS and without 2ME) by sodium dodecyl sulfate-polyacrylamide gel electrophoresis (SDS-PAGE, 12%). The concentration of the protein was determined using Bradford’s assay by a spectrophotometer (Eppendorf, Germany). To confirm the purified recombinant proteins, Western blot assay was carried out by standard protocols using anti-His antibody (Penta His Antibody; Qiagen) and with *P. falciparum*-infected human sera that reacted with rPfAMA-1 under both reducing and non-reducing conditions. Protein migration at different sizes on SDS-PAGE in the presence and absence of 2ME indicates the presence of a disulfide bound, suggesting that all recombinant proteins had a tertiary shape in their antigens.

### Expression and purification of recombinant PfMSP-1_19_ (rPfMSP-1_19_)

rPfMSP-1_19_ was expressed as described previously [11]. Briefly, rPfMSP-1_19_ protein was expressed in *E. coli* BL21. Overnight cultures from single colonies of PfMSP-1_19−_ specific *E. coli* were expanded in TB (pH 7.2) containing ampicillin (100 μg/ml) at 37°C with shaking (150 rpm) until an OD of 0.6-0.7 at 600 nm was reached. In addition, the expression of GST-PfMSP-1_19_ was induced with 0.5 mM IPTG (Sigma, USA). The culture was further grown for 4 h, and the *E. coli* cells were harvested by centrifugation and kept in −70°C until use. The cell pellet was dissolved in PBS 1× (pH 7.4) and lysed on ice with 10 sonication cycles (Ultraschall-Prozessor, Germany), each consisting of 20-s pulses at 20-s intervals. The bacterial lysate was centrifuged at 14,000 × g at 4°C for 30 min. The supernatant was incubated with glutathione 4B Sepharose resin (Amersham Biosciences, USA) at 4°C for 1 h, and the resin was packed into a column. The column was washed with PBS 1×, pH 7.4 (10-column volume). The bound protein was eluted with a buffer, containing 50 mM Tris–HCl and 10 mM reduced glutathione, pH 8. The fractions containing PfMSP-1_19_ were desalted with Econo-Pac 10 DG columns (BioRad, USA) according to the manufacturer’s manual and then concentrated with a concentrator (Eppendorf, Germany). The eluted proteins were analysed under reducing (with 1% SDS and 2% 2ME) and non-reducing conditions (with SDS and without 2ME) by SDS-PAGE (12%), and the concentration of the protein was determined using Bradford's assay by a spectrophotometer (Eppendorf, Germany).

### Mice immunization

Inbred BALB⁄c female mice (6–8 weeks old) were obtained from Laboratory Animal Science Department, the Institut Pasteur Iran. Mice groups (n = 7) were immunized subcutaneously at the base of tail with 30 μg and 35 μg of the rPfAMA-1and rPfMSP-1_19_, respectively. In priming and boosting, the antigens were then emulsified in complete Freund's adjuvant (CFA, Sigma, St. Louis, MO, USA) and with incomplete Freund's adjuvant (IFA, Sigma) in 1:1 ratio. The mice control groups were immunized with PBS alone, PBS in Freund's adjuvant and rGST alone. The animals were boosted on days 14 and 28 and bled on days 0 (pre-immune), 21, and 35 of first immunization.

### Indirect immunofluorescence antibody test (IFAT)

IFAT assay was performed to test the ability of the anti-PfAMA-1 and anti-PfMSP-1_19_ sera of immunized mice, to recognize the native form of both antigens on merozoite surface and to determine the similarity between epitopes in recombinant forms and corresponding native proteins. For this purpose, multispot slides of parasites were prepared from *P. falciparum* culture, air-dried and then fixed in cold acetone for 10 min. Polyclonal mouse sera diluted (1:100–1:12,800) in PBS were added to the spots and incubated in a wet chamber for 60 min. After washing three times with PBS (pH 7.4), each well was covered with 20 μL of the fluorescein-conjugated anti-mouse polyvalent IgG (1:40) and then left in a wet chamber for 40 min. Again, after washing three times with PBS, coverslips were placed on each slide and examined under a fluorescence microscope (Nikon E200, Tokyo, Japan) with an oil immersion objective (100×). The serum samples obtained from normal mice were used as negative controls.

### Comparative analysis of ELISA assays using single or combined rPfAMA-1 and rPfMSP-1_19_

In the present study, IgG antibody responses of individuals during acute infection to rPfAMA-1 and rPfMSP-1_19_ antigens (as single or in combination) were measured by an ELISA as described previously with some modifications [11]. In brief, Maxisorp flat-bottomed 96-well microplates (Grainer, Labortechnic, Germany) were coated duplicate with 200 ng of either rPfAMA-1 or rPfMSP-1_19_ and in combination of rPfAMA-1and rPfMSP-1_19_ (100 ng and/or 200 ng of each antigen/well) or GST alone (as control) in 0.06 M carbonate-bicarbonate buffer (pH 9.6) and then incubated at 4°C overnight. The plates were washed with PBS-Tween (PBS-T) and blocked with bovine serum albumin (BSA)-PBS-0.05% Tween. Serum was added in duplicate at a dilution of 1:200 (in BSA-PBS-0.05% Tween, 100 μl/well). After washing with PBS-T, the plates were incubated with horseradish peroxidase-conjugated goat anti-human IgG (Sigma, USA) at 1:35,000 concentration. Finally, the enzyme reaction was developed with o-phenylediamine dihydrochloride-H_2_O_2_ (OPD, Sigma, USA) and stopped with 2 N H_2_SO_4_. The OD was measured using an ELISA microplate reader (Biotech, USA) at 490 nm. All samples were re-tested if there was a discrepancy of greater than 20% between the duplicates. Standardization of the plates was achieved using positive-control serum pools on each plate. Background (determined from the wells with either no serum or GST) was subtracted from the mean of each sample, and a cut-off value was determined as the mean plus three standard deviations from the 30 negative control serum samples which were included in each assay.

### Statistical analysis

A database was generated with SPSS 20.0 for windows (SPSS Inc., USA). As the antibody levels were not normally distributed, non-parametric tests were used. Differences in the proportions of IgG-positive subjects were assessed using the McNemar's test or Chi square comparison of proportions as appropriate. Furthermore, differences between the mean absorbance of antigens alone or in combination were analysed by using Wilcoxon Signed Ranks test or Friedman test as appropriate. The Spearman’s correlation test was also used to assess the association between antibody levels with age as well as exposure. *P* values < 0.05 were considered statistically significant. The sensitivity of each test was measured by dividing the number of positive IgG sera to total numbers of sera obtained from *P. falciparum*-infected individuals.

## Results

### Detection of *P. falciparum* parasites by nested PCR

Based on both microscopy and nested-PCR results, all 101 patients were shown to be infected with *P. falciparum,* as a mono-infection, and none of the healthy control individuals had either *P. falciparum* or *P. vivax* infection.

### Recognition of native PfAMA-1 and PfMSP-1_19_ on the surface of *P. falciparum* merozoite by mice polyclonal antibodies to rPAMA-1 and rPfMSP-1_19_

**Figure 1:**
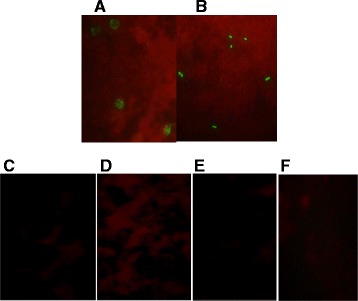
**IFAT for recognition of native form of AMA-1 and MSP-1**_**19**_ **on the** ***P. falciparum*** **parasites with polyclonal antibodies induced in mice.** Green fluorescence is visible due to the recognition of surface AMA-1 and MSP-1_19_ on *P. falciparum* merozoite by sera of the mice immunized with **(A)** rPfAMA-1+ CFA/IFA (n = 7); **(B)** rPfMSP-1_19_ + CFA/ICFA (n = 7); **(C)** GST + CFA/ICFA (n = 7); **(D)** PBS + CFA/ICFA (n = 7); **(E)** PBS (n = 7); **(F)** normal mouse sera (n = 7). CFA: Complete Freund's adjuvant; IFA: Incomplete Freund's adjuvant.

Anti-rPfAMA-1 and -rPfMSP-1_19_ produced in mice recognized the native protein present on the surface of *P. falciparum* merozoite at late schizont (or merozoite) stage with high intensity, as indicated by the grape-like fluorescence pattern (Figure 1A and B). Moreover, none of the control mice sera recognized the native protein on *P. falciparum* parasite (Figure 1C, D, E, and F), confirming that there are common epitopes in recombinant forms that correspond to native proteins. The reduced and non-reduced SDS-PAGE as well as Western blot analysis also confirmed that rPfAMA-1 (~55 kDa) and rPfMSP-1_19_ (37.5 kDa) proteins had proper conformation and folding.

### Antibody responses to rPfAMA-1 antigen

**Figure 2:**
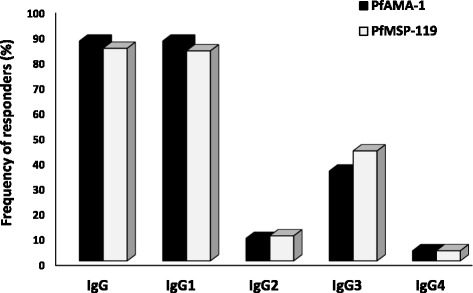
**Prevalence of IgG and its subclass responses to rPfAMA-1 and rPfMSP-1**_**19**_ **antigens among individuals with** ***P. falciparum*** **patent infection (n = 101) from malaria-endemic area of Iran.** No significant difference was observed in the proportions of IgG, IgG1, IgG2, IgG3, and IgG4 responses to PfAMA-1 and PfMSP-1_19_ antigens (*P* > 0.05; McNemar's test). The cut-off values of the controls were as follow: IgG: 0.284, IgG1: 0.273, IgG2: 0.2, IgG3: 0.37, and IgG4: 0.25 for PfAMA-1 antigen; IgG: 0.323, IgG1: 0.319, IgG2: 0.215, IgG3: 0.357, and IgG4: 0.2 for PfMSP-1_19_ antigen.

PfAMA-1 was expressed in *E. coli* M15-pQE30, and the purified protein was analysed by SDS-PAGE with a molecular mass of ~55 kDa. The purity of the recombinant proteins was evaluated by Western blot assay. The result showed that expressed proteins migrated at different sizes in the presence and absence of 2ME contained a disulfide bound. Total IgG antibody responses to PfAMA-1 was determined in 101 individuals (aged 4 to 75 years; median = 27 years; Table 2) and only 87.1% (88/101) had positive IgG antibody responses to rPfAMA-1 antigen (Figure 2). None of the sera from healthy individuals (control group) contained IgG antibody to rPfAMA-1, which confirms the specificity of the present results.

**Table 2:** **Comparative analysis of the level of IgG and subclass antibodies to rPfAMA-1 and rPfMSP-1**_**19**_ **antigens among individuals with patent** ***P. falciparum*** **infection from south-eastern Iran**

**Antigen**	**Mean OD**_**490**_ **± SD**				
	**IgG**	**IgG1**	**IgG2**	**IgG3**	**IgG4**
**PfAMA-1**	1.096 ± 0.393	1.307 ± 1.001	0.232 ± 0.027	0.938 ± 0.436	0.502 ± 0.144
Frequency of responders (%)	88 (87.1)	88 (87.1)	9 (8.9)	36 (35.6)	4 (4)
Cut-off	0.284	0.273	0.2	0.37	0.25
**PfMSP-1**_**19**_	1.467 ± 0.619	1.654 ± 0.802	0.404 ± 0.191	0.936 ± 0.430	0.467 ± 0.088
Frequency of responders (%)	85 (84.2)	84(83.2)	10 (9.9)	44 (43.6)	4 (4)
Cut-off	0.323	0.319	0.215	0.357	0.2
*P* value (Spearman’s correlation test)	**<0.0001**	**< 0.0001**	**< 0.0001**	**< 0.0001**	**< 0.0001**

The mean OD_490_ nm value was considered as a measure of the anti-PfAMA-1 or -PfMSP-1_19_ -specific antibody responses of each serum sample by ELISA assay. The cut-off values were calculated by mean ODs of normal human sera (n = 30) out of malaria- endemic areas plus 3 standard deviation (SD). There was a significant correlation among the mean ODs of IgG, IgG1, IgG2, IgG3, and IgG4 to PfAMA-1 and PfMSP-1_19_ antigens (IgG, *r* = 0.603, *P* <0.0001, IgG1, *r* = 0.593, *P* < 0.0001; IgG2, r = 0.433, *P* < 0.0001; IgG3, *r* = 0.457, *P* < 0.0001; and IgG4, *r* = 0.464, *P* < 0.0001; Spearman’s correlation test).

### IgG subclass response to rPfAMA-1 antigen

Serum samples positive for total anti-rPfAMA-1 IgG were evaluated for IgG subclass responses to rPfAMA-1 antigen. In individuals who were infected with *P. falciparum*, the IgG1 to rPfAMA-1 (87.1%, OD_490_ = 1.307, Figure 2 and Table 2) was the dominant subclass. The second frequent subclass was IgG3 (35.6%, OD_490_ = 0.938, Figure 2 and Table 2). In case of IgG2 and IgG4, the frequency distribution of individuals that recognized the antigen was 8.9% and 4% (mean OD_490_ = 0.232 and 0.502, respectively; Figure 2 and Table 2). Furthermore, heterogeneity in IgG1 and IgG3 isotype responses was observed (Table 3). The results indicated that IgG1 and IgG3 were predominant over IgG2 and IgG4 antibodies. In addition, there were significant differences among the levels of IgG, IgG1, IgG2, and IgG4 antibodies (Friedman test, *P <* 0.05).

**Table 3:** **The heterogeneity in IgG1 and IgG3 responses to rPfAMA-1 and rPfMSP-1**_**19**_ **antigens in individuals with patent** ***P. falciparum*** **infection, living in Chabahar district of Iran**

**Antigen**	**IgG1** ^**+**^ **/IgG3** ^**+**^ **no. (%)**	**IgG1** ^**+**^ **/IgG3** ^**−**^ **no. (%)**	**IgG1** ^**−**^ **/IgG3** ^**+**^ **no. (%)**	**IgG1** ^**−**^ **/IgG3** ^**−**^ **no. (%)**	***χ*** ^**2**^ **test** ***P*** **value**
PfAMA-1	36 (35.6)	52 (51.5)	0	13 (12.9)	= 0.357
PfMSP-1_19_	43 (42.6)	41(40.6)	1 (0.99)	16 (15.8)	

### Antibody responses to rPfMSP-1_19_ antigen

PfMSP-1_19_ was expressed in *E. coli* BL21-pGEX-KG, and the purified protein was analysed by SDS-PAGE with a molecular mass of ~37.5 kDa. The purity of the recombinant protein was evaluated by Western blot assay. The result showed that the expressed PfMSP-1_19_ migrated as a single homogeneous band on SDS-PAGE under non-reducing conditions, indicating that it is largely composed of a single conformer. Total IgG antibody responses to rPfMSP-1_19_ was determined in 101 individuals (aged 4 to 75 years; median = 27 years; Table 1) and only 84.2% (85/101) had positive IgG antibody responses to rPfMSP-1_19_ antigen (Figure 2). None of the sera from healthy individuals (control group) contained IgG antibodies to PfMSP-1_19_, which confirms the specificity of the present results.

### IgG subclass response to rPfMSP-1_19_ antigen

Serum samples positive for total anti-PfMSP-1_19_ IgG were evaluated for IgG subclass responses to rPfMSP-1_19_ antigen. In individuals who were infected with *P. falciparum*, the IgG1 to rPfMSP-1_19_ (83.2%, OD_490_ = 1.654, Figure 2 and Table 2) was the dominant subclass. The second frequent subclass was IgG3 (43.6%, OD_490_ = 0.936, Figure 2 and Table 2). In case of IgG2 and IgG4, the frequency distribution of individuals that recognized the antigen was 9.9% and 4% (mean OD_490_ = 0.404 and 0.467, respectively; Figure 2 and Table 2). Furthermore, heterogeneity in IgG1 and IgG3 isotype responses of individuals was observed (Table 3). The results indicated that IgG1 and IgG3 were predominant over IgG2 and IgG4 antibodies. In addition, there were significant differences among the levels of IgG, IgG1, IgG2, IgG3, and IgG4 antibodies (Friedman test, *P <* 0.05).

### Exposure- and age-dependent IgG, IgG1, and IgG3 responses

The levels of IgG, IgG1, and IgG3 antibodies to rPfAMA-1 were not correlated with exposure (*r =* 0.061, *P* = 0.544 for IgG; *r =* 0.042, *P* = 0.673 for IgG1, and *r =* 0.014, *P* = 0.893 for IgG3; Spearman’s correlation test) or age (*r =* 0.082, *P* = 0.416 for IgG; *r =* 0.060, *P* = 0.550 for IgG1, and *r =* 0.172, *P* = 0.085 for IgG3; Spearman’s correlation test). Likewise, the levels of IgG, IgG1, and IgG3 antibodies to the rPfMSP-1_19_ were not correlated with exposure (*r =* 0.104, *P* = 0.302 for IgG; *r =* 0.013, *P* = 0.895 for IgG1, and *r = −*0.022, *P* = 0.826 for IgG3; Spearman’s correlation test) or age (*r =* 0.128, *P* = 0.2 for IgG; *r =* 0.078, *P* = 0.435 for IgG1, and *r =* 0.118, *P* = 0.242 for IgG3; Spearman’s correlation test).

**Figure 3:**
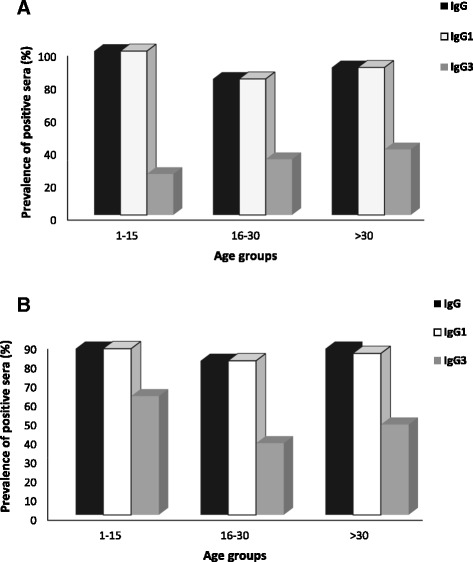
**Association between age and IgG, IgG1, and IgG3 antibody responses to rPfAMA-1 (A) and rPfMSP-1**_**19**_ **(B) antigens.** The prevalence of positive responders for IgG, IgG1, and IgG3 antibodies for each age group is shown in the Figure. In the different age groups, no significant difference was observed in the prevalence of responders to PfAMA-1 and PfMSP-1_19_ antigens for IgG, IgG1, and IgG3 (*P >* 0.05, *X* ^*2*^ test). Age groups are: 1–15 years (n = 8), 16–30 years (n =53), and ≥ 31 years (n = 40).

**Figure 4:**
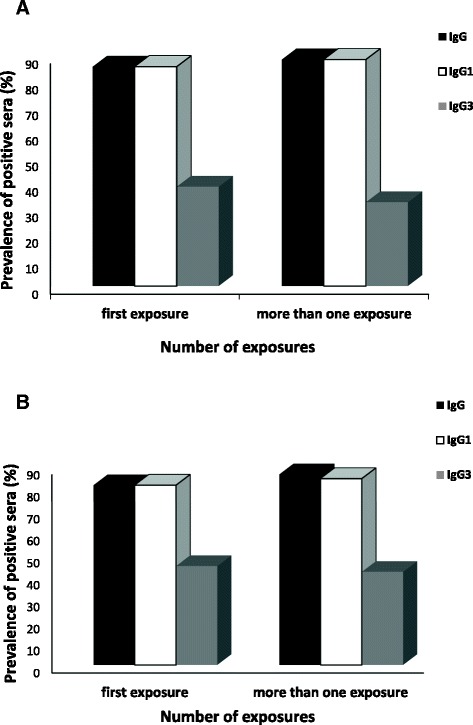
**Association between episodes of** ***P. falciparum*** **infection and IgG, IgG1, and IgG3 antibody responses to PfAMA-1 (A) and PfMSP-1**_**19**_ **(B) antigens.** In the individuals with different malaria episodes (*P >* 0.05, *X* ^*2*^ test), no significant difference was observed in the prevalence of responders to PfAMA-1 and PfMSP-1_19_ antigens for IgG, IgG1, and IgG3. The groups are: (i) no previous malaria episodes (n = 49) and (ii) individuals with one or more previous malaria episodes (n = 52).

Regarding the analysis of the correlation between the frequency of IgG antibodies and age, the sera of the 101 individuals were separated into three groups: (i) 1–15 years old (n = 8), 16–30 years old (n = 53), and ≥ 31 years old (n = 40). No significant difference was found in the prevalence of positive sera for PfAMA-1 and PfMSP-1_19_-specific IgG, IgG1 and IgG3 antibodies in different age groups (Chi-square, *P* > 0.05, Figure 3A and B), indicating that antibody responses against PfAMA-1 and PfMSP-1_19_ were not correlated with age. It was then determined whether there was a correlation between the frequency of IgG antibodies and episodes of *P. falciparum* infection. For this purpose, the sera of the 101 individuals were separated into two groups: (i) primary infected, individuals with no previous malaria episodes (n = 49) and (ii) individuals with one or more previous malaria episodes (n = 52). The frequency of responders to both antigens did not change significantly when they divided in primary and multiple-infected (Chi-Square test, *P >* 0.05) confirming that antibody response against PfAMA-1 and PfMSP-1_19_ was established after a single exposure to malaria (Figure 4A and B). This result shows that specific IgG responses to both antigens are developed after even one malaria episode.

### Comparative analysis of the naturally acquired antibody responses to rPfAMA-1 and rPfMSP-1_19_ antigens

**Figure 5:**
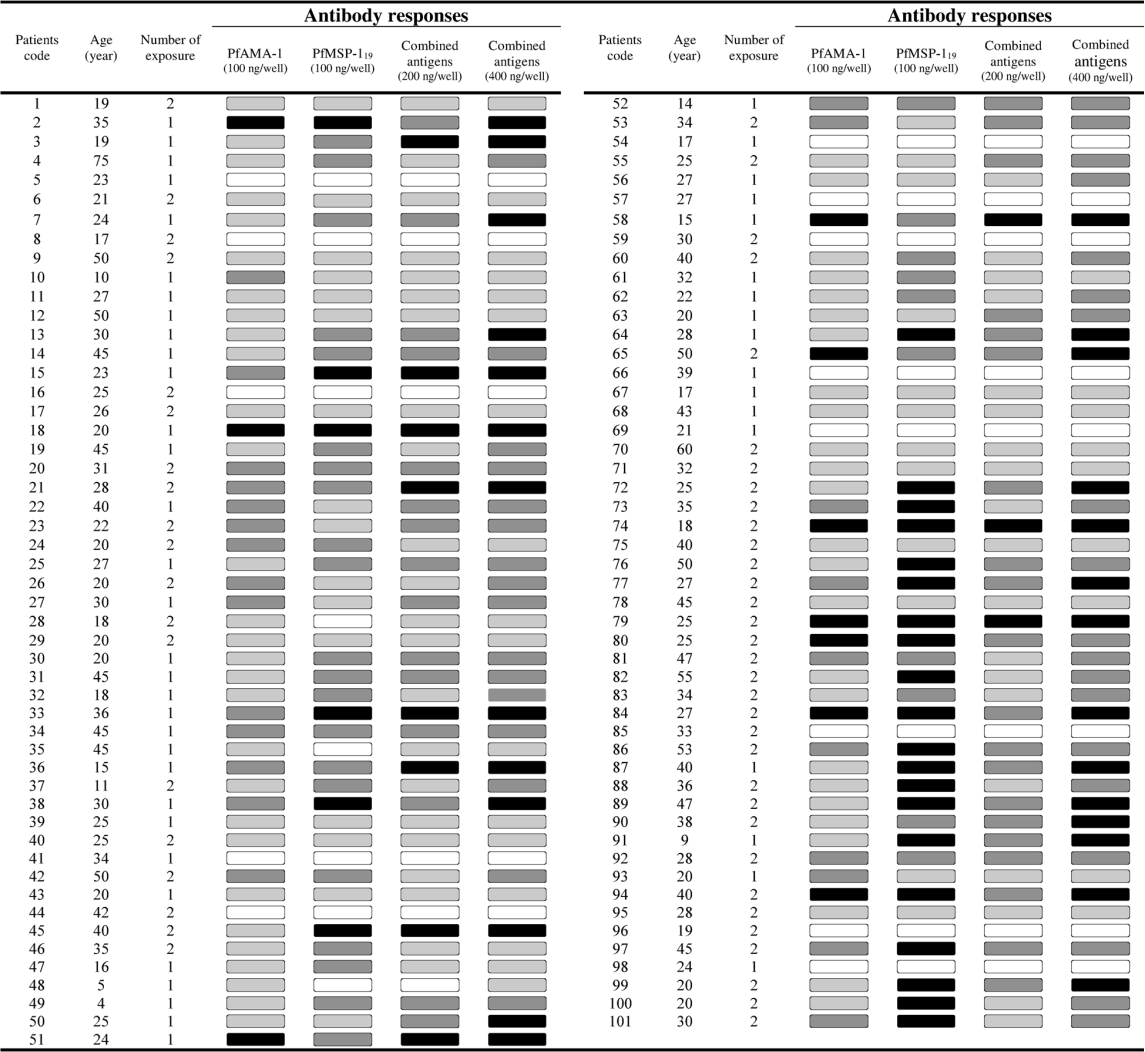
**Pattern of total IgG responses to single rPfAMA-1, rPfMSP-1**_**19**_ **, and combined antigens in individuals infected with** ***P. falciparum*** **(n = 101).** Ages are given in years. Cut-off values were 0.284, 0.323, 0.351, and 0.36 for PfAMA-1, PfMSP-1_19_, combined antigens with 100 ng of each and 200 ng of each, respectively. The OD mean values have been divided into the following groups: OD > 1.5: High-positive antibody responses (black). 1 < OD < 1.5: Medium-positive responses (dark gray), OD < 1: Low-positive responses (pale gray), and OD < Cut-off: Negative (white).

**Figure 6:**
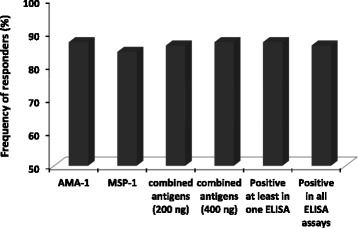
**Prevalence of tested samples with positive IgG antibody responses to either rPfAMA-1 and rPfMSP-1**_**19**_ **antigens alone or in combination (200 and 400 ng of antigens in equal ratio).**

The frequency of individuals with IgG antibodies to combined antigens (100 ng of each antigen/well) was only 86.2% during patent infection with *P. falciparum*. No response to either antigen was observed in 13.8% of the individuals (Figures 5 and 6; Table 4). The difference in the prevalence of anti-PfAMA-1 and -PfMSP-1_19_ IgG responses in the tested samples was not statistically significant (with mean OD_490_ = 1.096 ± 0.393 and 1.467 ± 0.619; cut-offs 0.284 and 0.323, respectively; *P =* 0.25, McNemar's test; Table 4). None of the sera from control groups contained anti-PfAMA-1 and/or -PfMSP-1_19_ IgG antibodies. The present data confirmed that the two antigens were immunogenic during natural infections. However, when rPfAMA-1 and rPfMSP-1_19_ were combined at equal ratios of 200 ng (100 ng each antigen/well) and 400 (200 ng each antigen/well), sero-positivity of 86.2% and 87.1% were obtained, respectively (Figure 6, Table 4). Interestingly, 3% (3/101) of the tested samples had positive IgG antibody responses to rPfAMA-1 but not to rPfMSP-1_19_ antigens, indicating the higher sensitivity of rPfAMA-1 than rPfMSP-1_19_ (Figures 5 and 6, Table 4). There was also statistically difference among the mean absorbance of antibodies to both antigens when used in ELISA either alone or in combination (*P <* 0.05, Friedman test). However, when the mean absorbance was compared in paired groups, no significant difference was observed in the mean absorbance of antibodies to combination of rPfAMA-1 and rPfMSP-1_19_ at concentration of either 200 ng or 400 ng (*P* > 0.05, Wilcoxon Signed Ranks test).

**Table 4:** **Prevalence of IgG responses to rPfAMA-1, and rPfMSP-1**_**19**_ **alone and in combination antigens in 101 samples of patients who were infected with** ***P. falciparum***

**Antigen**	**Negative sera No. (%)**	**Positive sera No. (%)**	**Mean OD ± SD of positive samples**	**Cut-off value**
PfAMA-1	13 (12.9%)	88 (87.1%)	1.096 ± 0.393	0.284
PfMSP-1_19_	16 (15.8%)	85 (84.2%)	1.467 ± 0.619	0.323
Combined antigens (200 ng/well)	14 (13.8%)	87 (86.1%)	1.133 ± 0.379	0.351
Combine antigens (400 ng/well)	13 (12.9%)	88 (87.1%)	1.390 ± 0.437	0.351

## Discussion

In elimination programmes, reduction in disease and death due to *P. falciparum* is the primary objective. Although the prospects of achieving global malaria eradication have been diminished by the limited available intervention tools, vaccines could be considered effectively in these programmes; for instance, they have also vital role in global eradication of smallpox and the elimination of Poliomyelitis from the world. Moreover, for vaccine development, sero-epidemiology studies on various populations with a different genetic background and endemicity in malaria settings would help to understand the host immune responses to malaria parasites as well as the endemicity of the disease. Besides, in malaria elimination programmes, monitoring changes in transmission intensity and identification of residual foci of malaria by using sensitive and reliable tools is very important for measuring the success of the programme. Therefore, the goal of this study was to compare and analyse the naturally acquired antibody responses to two recombinant proteins representing two asexual erythrocytic stage of *P. falciparum* (PfAMA-1 and PfMSP-1_19_) by human IgG antibodies among naturally exposed individuals living in the malaria hypoendemic setting, Iran. These two proteins not only are the asexual erythrocytic stage vaccine candidates but also are serological markers particularly useful for detection of relative antibody responses in areas of low endemicity [53,58].

In the present investigation, both antigens were produced in *E. coli*, and the results showed that both expressed proteins were folded correctly and suitable for ELISA. The value of using recombinant protein in vaccine development against malaria has been also shown in earlier studies. These surveys demonstrated that the cysteine-rich sequence of PfMSP-1_19_ with two epidermal growth factor (EGF)-like motifs [59] could be expressed in *E. coli* in a correctly folded manner [46], and that it plays an important role in the induction of protective immunity [60]. In addition, the protection elicited by AMA-1 is directed at epitopes dependent on the disulfide bonding [61] located in the AMA-1 ectodomain; hence, the correct conformation is critical for AMA-1 based vaccine development.

In elimination and eradication strategies, understanding the immunity to malaria parasites is crucial for successful and reliable interventions. The Iranian government aims to eliminate this poverty-related disease from malaria-endemic settings, mostly close to Pakistan and Afghanistan border areas, where both *P. vivax* (>80%) and *P. falciparum* (<20%) are prevalent. In the present work, the interaction between the host immune system and parasites showed that 87.1% and 84.2% of the studied individuals had positive anti-PfAMA-1 and -PfMSP-1_19_ IgG antibody responses, respectively, suggesting that both of these expressed antigens are well-recognized asexual-stage parasite antigens. Although the present finding documents that the frequencies of antibodies to both recombinant antigens are almost similar in the areas of unstable transmission, the role of these antibodies in protection to malaria needs further study. However, the absence of such response in about 13% of the individuals could be perhaps explained by unknown human genetic factors [62] and/or the first or short exposure to these antigens, which may be insufficient to induce considerable immune responses. This slow development of naturally acquired malaria immunity has been shown by others in low to moderate malaria transmission settings [63,64].

Analysis of IgG isotype response to the PfAMA-1 and PfMSP-1_19_ antigens is important for evaluating protective activity as IgG subclasses differ in their immune effector functions and having such knowledge is important for understanding the immunity to vaccine development. The result of the present study confirmed previous studies [42,54,65,66] that showed IgG1 and IgG3 isotypes were the predominant subclasses in response to both antigens. These subclass responses might perhaps relate to antigen properties, number of exposure, host age, and genetic determinants. The high prevalence of anti-PfMSP-1_19_ IgG1 responses among studied individuals was in contrast to what was reported from Senegalese adults (in Dielmo and Ndiop) that a greater proportion of individuals were anti-MSP-1 IgG3 positive [67]. This finding was in line with earlier report of very little or no IgG3 to PfAMA-1 [66].

In addition, the present result was in contrast to the previous report that stated the frequency of PfMSP-1_19_-specific IgG1 was higher among subjects (living in different areas of Brazil) with a long-term exposure to malaria, as compared to the subjects sporadically-exposed [68]. In this study, although anti-PfAMA-1 and -PfMSP-1_19_ IgG1 was predominant, there was also a mixed IgG1/IgG3 response as reported by others [69]. This heterogeneity in IgG1 and IgG3 recognition could be related to either different epitopes in PfAMA-1 antigen recognized by these two IgG subclasses or short half-life of IgG3 antibody in the serum sample. Moreover, it is well established that IgG1 and IgG3 subclasses mediate opsonization and complement fixation of pathogens, and they are involved in antibody-mediated protective immunity against Plasmodium blood stages [70,71]. Therefore, the finding that IgG subclasses to both antigens are mainly of the IgG1/IgG3 type with high frequencies indicate that this high prevalence might be associated with protective effect on cell-mediated mechanisms from falciparum malaria as shown by others [15,42,65-75].

As the interaction between the host immune system and parasites differs based on the degree of the malaria endemicity, it has been suggested that in malaria high-endemic areas, the acquisition of natural immunity to *P. falciparum* requires several years of uninterrupted exposure [68,76]. However, in hypoendemic or mesoendemic areas, there is no association between age and exposure to malaria [7-9]. In the present study, in the unstable and low malaria transmission, the frequency of responders to PfAMA-1 and PfMSP-1_19_ was not correlated with either age or number of exposure to malaria, which confirms the previous reports [7-9] and indicates that PfAMA-1 and PfMSP-1_19_ are highly immunogenic during natural human infections. The present result was also in agreement with the result of previous studies in low-endemic areas of Senegal [32] and West Africa [41,77], where no such correlation was observed. Nevertheless, a statistically significant age-related change in antibody levels to PfAMA-1 [78] and PfMSP-1_19_ [79] was observed in the previous studies.

In the advanced phases of malaria elimination programmes, the technique for assessment of malaria transmission intensity and evaluation of interventions during this effort are highly required. Recently, there has been a recall for elimination of malaria with the scaling-up interventions; therefore, malaria burden and transmission declined across a number of countries [80-82]. In such situations, serological techniques using reliable markers could be applied for detecting and targeting clusters of infection, to reduce the local parasite reservoir and interrupt transmission [83]. In this study, the applicability of using two serological markers to detect sero-positive cases in such an unstable, hypoendemic, and low transmission settings, where the sensitivity of parasite prevalence surveys is limited, was tested. Each antigen was highly specific and reactive to the tested sera, and the sensitivity of a single antigen for detection was similar with that of the two combined antigens. Nevertheless, since 3% of samples were positive for PfAMA-1 but negative for PfMSP-1_19_, the present study is in favour of using multiple antigens for antibody-based detection in this area and other similar settings during elimination programmes.

## Conclusion

In summary, the present results suggest that the two tested recombinant antigens are immunogenic molecules and useful tools to perform immuno-epidemiological studies in low transmission areas of Iran. These data also provide, for the first time, information on the characteristics of naturally acquired immunity in populations exposed to malaria transmission in Iran. Indeed, it could be beneficial for development and testing of a PfAMA-1 and PfMSP-1_19_-based vaccine in Iran, where malaria is endemic. This study specially demonstrates high level frequencies of antibodies to rPfAMA-1 and rPfMSP-1_19_ among individuals infected with *P. falciparum* in areas of unstable and low transmission, indicating that these two expressed antigens could be used in combination as serology markers during elimination campaigns in this region.
